# Exposure to PM_2.5_ affects blood lipid levels in asthmatic rats through notch signaling pathway

**DOI:** 10.1186/s12944-019-1102-8

**Published:** 2019-08-07

**Authors:** Tianrong Zhang, Yan Zheng, Yizhen Gao, Tianyang Zhao, Shuangyu Guo, Liwei Yang, Yanbin Shi, Liting Zhou, Lin Ye

**Affiliations:** 1 Department of Occupational and Environmental Health, School of Public Health, Jilin University, 1163 Xin Min Street, Changchun, 130021 China; 2 The Department of Cadre ward, the first Hospital of Jilin University, Changchun, Jilin, 130021 China

**Keywords:** Atmospheric PM_2.5_, Asthma, Notch signaling pathway, Dyslipidemia

## Abstract

**Background:**

Epidemiological studies have confirmed atmospheric PM_2.5_ could affect asthma, and dyslipidemia may be related to pathogenesis of asthma. Recent studies show Notch ligands had lipid combination domains which are responsible for regulating lipid levels. However, the effect of PM_2.5_ on asthmatic rats’ lipid levels and the role of Notch signaling pathway is unclear.

**Methods:**

Rats were treat with ovalbumin (OVA) to establish asthma models. Notch signaling pathway inhibitor (DAPT) was injected intraperitoneally. Asthmatic and healthy rats were exposed to different concentrations of PM_2.5_. Lung tissues were collected and the expression of Hes1 protein was detected by Western Blot. Blood samples were collected to detect the serum lipid levels.

**Results:**

Hes1 expression levels in healthy and asthma pathway inhibition groups were lower than those in control groups. Compared with control group, rats exposed to PM_2.5_ in middle and high dose, the levels of TG and TC were decreased. Similar results were observed after exposure to the same concentration of PM_2.5_ in asthmatic rats. Rats, which were exposed to PM_2.5_ after being established the asthma model successfully, could exhibit more significant dyslipidemia than those with direct exposure. After Notch signaling pathway inhibited, TC and LDL in asthma pathway inhibition group were lower than those in healthy group.

**Conclusions:**

PM_2.5_ can affect the lipid levels of asthmatic rats through the Notch signaling pathway.

## Background

Fine specific matter (PM_2.5_) refers to particles with an aerodynamically equivalent diameter ≤ 2.5 μm. Researches had indicated that PM_2.5_ was a risk factor for the development of many diseases, including metabolic syndrome. Exposure to PM_2.5_ can cause the accumulation of triglycerides (TG) and total cholesterol (TC) in the serum, which may lead to dyslipidemia by triggering inflammation [1]. X. Wang and Z. Zheng point out PM_2.5_ exposure causes lung and vascular abnormalities, possibly related to oxidative stress, endoplasmic reticulum stress and inflammatory response-related signaling pathways [2–4]. Meanwhile, it has been reported that PM_2.5_ exposure causes severe inflammatory reactions leading to hypothalamic injury, which is related to energy balance and metabolic changes [2].

Asthma is an airway chronic inflammatory disease characterized by inflammatory infiltration in the bronchial wall and excessive secretion of mucus [5]. There is increasing evidence that dyslipidemia and changes in the endocrine function of adipose tissue may exacerbate asthma [6, 7]. An epidemiological survey found higher incidence of dyslipidemia in asthmatic patients than that in non-asthmatic patients [8]. Garmendia JV pointed out that metabolic syndrome is a syndrome involving dyslipidemia, insulin resistance, obesity and/or hypertension. Patients with metabolic syndrome have a higher risk of developing severe asthma [9]. Husemoen LL pointed obesity and insulin resistance was associated with asthma. Insulin resistance leads to excess circulating nutrients, then producing excess of triglycerides, free fatty acids and glucose, which can disrupt metabolism, increase inflammation and exacerbate asthma [10]. Peng J found there is a significant association between asthma and the serum levels of high density lipoprotein (HDL) and low density lipoprotein (LDL) [11]. Rastogi et al. reported that children with asthma have lower levels of HDL. The anti-inflammatory properties of HDL may be related to asthma [12]. Scichilone N found the levels of serum LDL have been implicated in the inflammatory cascade in a murine model of asthma [13].

Notch signaling is a complex pathway that regulates cell proliferation, differentiation, self-renewal, apoptosis and inflammatory responses [14–17]. It involves four Notch receptors and five ligands in the Jagged/Delta-like family. Notch receptors and ligands bind to each other, causing the release of the Notch intracellular domain (NICD). Translocation of NICD into the nucleus induces transcription of target genes such as Hes family [18]. Hes1 is the main effector of the Notch pathway [19]. At the same time, Notch signaling can regulate the terminal differentiation of eosinophils and influence the development of asthma [20]. Gamma-secretase is involved in the cleavage of the Notch protein, which is essential for Notch-mediated signaling. DAPT is a gamma-secretase inhibitor (GSI) that has been used to effectively block Notch signaling [21]. Studies have found that GSI inhibits eosinophil inflammatory responses may improve asthma symptoms [22]. Treatment of Apo E−/− mice with GSI reduces atherosclerotic symptoms by inhibition [23]. Recent studies have shown that Notch1 signaling is involved in the regulation of lipid synthesis and Notch1 signaling plays a conservative role in lipid metabolism [24].

Therefore, we speculate that PM_2.5_ may cause dyslipidemia in asthmatic rats through the Notch signaling pathway.

## Methods

### PM_2.5_ sampling and dry powder preparation

The large-flow PM_2.5_ environmental sampler and quartz fiber filter were used to continuously collect PM_2.5_ in the atmosphere throughout the main roads of Changchun City. After sampling, the filter membranes were dried, wrapped in aluminum foil, numbered, and stored at a low temperature. The cryopreserved membranes were cut into small pieces and placed in the bottom of a beaker. Then deionized water was added and the mixture was shaken by ultrasonic for 60 min. The particles on the filter were eluted and PM_2.5_ suspension was obtained. Six layers of sterile gauze were used to filter the PM_2.5_ suspension and the filtered suspension was dispensed and placed in a low temperature freezer at − 80 °C overnight. The sample was taken out and placed in a vacuum freeze dryer for freeze-drying treatment. After 12 h, the water completely evaporated. The dry gray floe was visible as the PM_2.5_ dry powder at the bottom of the bottle and stored at − 20 °C until use. School of Environment and Resources conducted source analysis.

### Selection of experimental animals

All experimental animals were purchased from the Experimental Animal Center of Jilin University (Changchun, China). One hundred and fourteen Wistar rats weighing 200-250 g were selected.

### Grouping of experimental animals

After 1 week of adaptive feeding, Wistar rats were randomly divided into three groups according to their sex ratio: the first group of 24 divided into 4 groups, 6 in each group, were blank control (BC) group, low dose (LD-PM_2.5_ 1.5 mg/kg) group, middle dose (MD-PM_2.5_ 7.5 mg/kg) group and high dose (HD-PM_2.5_ 37.5 mg/kg) group. The second group consisted of 40 rats divided into 5 groups of 8 in each group: blank control (BC) group, asthma model control (AC) group, asthma model low dose (AL-PM_2.5_ 1.5 mg/kg) group, asthma model middle dose (AM-PM_2.5_ 7.5 mg/kg) group and asthma model high dose (AH-PM_2.5_ 37.5 mg/kg) group. The third group consisted of 50 rats divided into 5 groups of 10 in each group: blank control (BC) group, healthy control (HC) group, healthy pathway inhibition (HI) group, asthma model control (AC) group and asthma pathway inhibition (AI) group.

### Establishment of asthma rat model

1. Sensitization for experimental rats for the first time: The OVA suspension was placed at a ratio of 1 mg OVA plus 100 mg Al(OH)_3_. After 1 week of adaptive feeding, rats were intraperitoneally injected with OVA at 1 ml/kg.
2. Intensifying sensitization of the experimental rats again: rats were intraperitoneally injected with OVA at 1 ml/kg again, so that rats are in a sensitive state.
3. The 21% aluminum hydroxide gel was formulated into a nebulizer with a concentration of 1% OVA, so that rats were in a 1% OVA atomization environment, fully sensitized, and onset of asthma in rats was induced. The atomization was made once every 2 days for 2 weeks.
4. Rat alveolar lavage fluid was drawn and stained to observe changes in inflammatory cells and evaluate whether the model was successful.

### PM_2.5_ exposure

After the first two groups were anesthetized with ether, 3 ml/kg body weight was infused into the PM_2.5_ physiological saline suspensions via the nasal cavity. The doses of the low, medium and high concentration group were 1.5, 7.5, and 37.5 mg/kg respectively, control groups were given physiological saline. PM_2.5_ were exposed once every 2 days and totally two times. Rats were treated 24 h after the last exposure. The third group of asthma model pathway inhibition group and healthy control pathway inhibition group were intraperitoneally injected with DAPT (0.5 mg/kg) twice. Asthma control group and healthy control group were intraperitoneally injected with the same volume of Dimethyl sulfoxide (DMSO), and the blank control group was given the same volume of normal saline. Except blank control group tracheal instillation of normal saline, the other four groups were nasally instilled with 7.5 mg/kg of PM_2.5_ physiological saline suspensions once every 2 days and two times in total. Rats were treated 24 h after the last exposure. The animal room temperature was kept at 23–25 °C and the humidity was maintained at 60–70%.

**Fig. 1:**
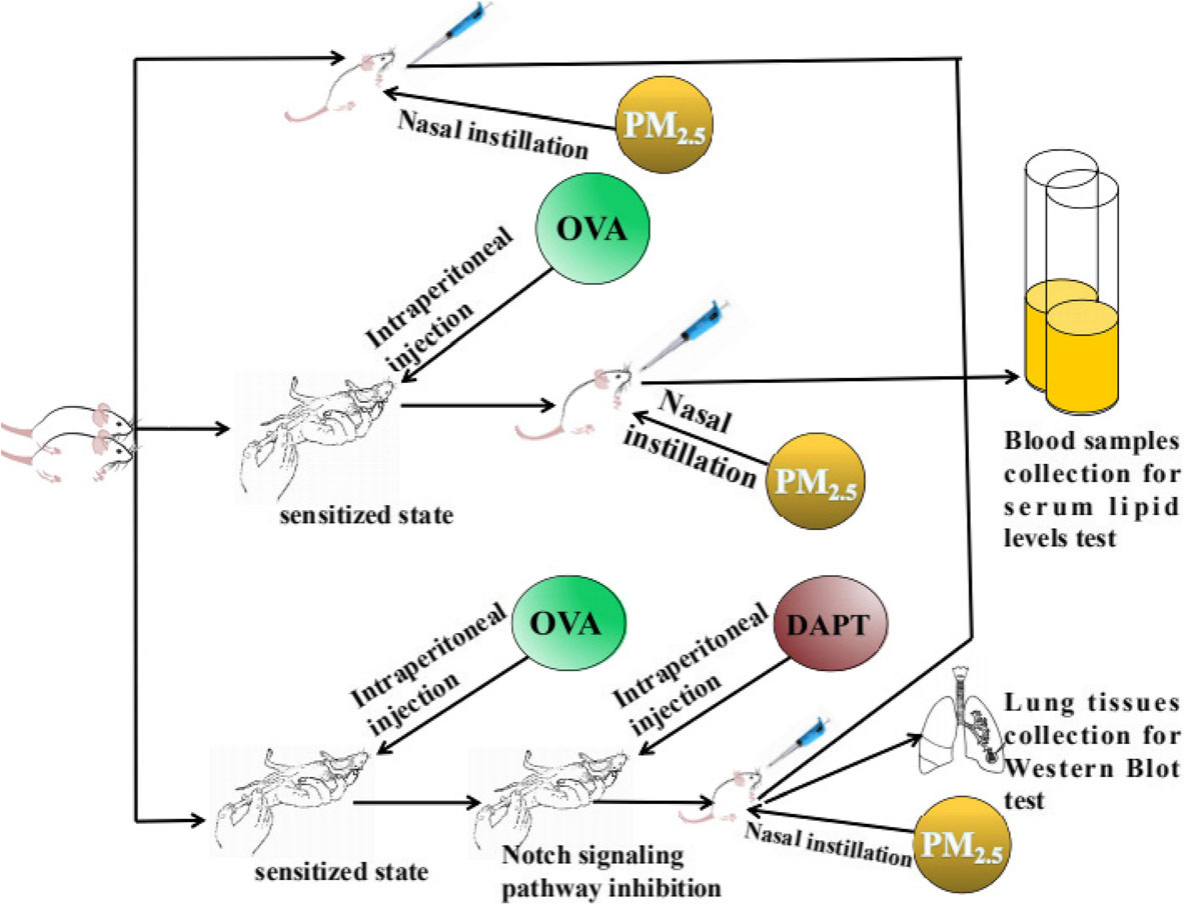
Figure flow of all experiments

The experimental flow was shown in Fig. 1.

### Tissue collection

After 4 weeks, the rats were weighed and anesthetized by 4% sodium pentobarbital in asepsis condition. Blood samples were drawn from the heart of each rat using vacuum blood collection tubes. The serum specimens were centrifuged at 2500 rpm for 20 min and then the sera were separated and stored at − 20 °C.Following the blood collection, lung was isolated and maintained in liquid nitrogen until protein extraction.

### Western blot

Determinations of Hes1 were assessed using western blot. Proteins were extracted from lung tissue. The concentration of protein samples was determined using the bicinchoninic acid protein assay kit (Beyotime, Shanghai, China). The proteins were separated by SDS-PAGE (Beyotime) and transferred to a nitrocellulose membrane (Pall, Ann Arbor,MI, USA). The membrane was then incubated in blocking solution with anti-Hes1 (Proteintech). Following the incubation of the secondary antibodies, enhanced chemiluminescence substrate (Beyotime) was then applied for 5 min prior to exposure.

### Detection of lipid in serum

According to the instructions provided by the manufacturer, the levels of TG in the serum were assayed using a GPO-PAP enzymatic method, HDL and LDL were assayed using a direct method, TC were assayed using a COD-PAP method (Nanjing Jiancheng Biotechnology, Nanjing, China).

### Statistical analysis

All data were tested by normality (Kolmogorov-Smirnov test) and homogeneity of variance (Levene’s test). Conditional data are presented as mean and standard error of mean (SE). We chose the t test for the comparison between the two groups and we performed one-way ANOVA on each parameter followed by a LSD’s post hoc test at each sampling time separately. Differences between groups were considered statistically significant if *p* < 0.05, which was two-sided.

## Results

### Asthmatic rat model evaluation

**Fig. 2:**
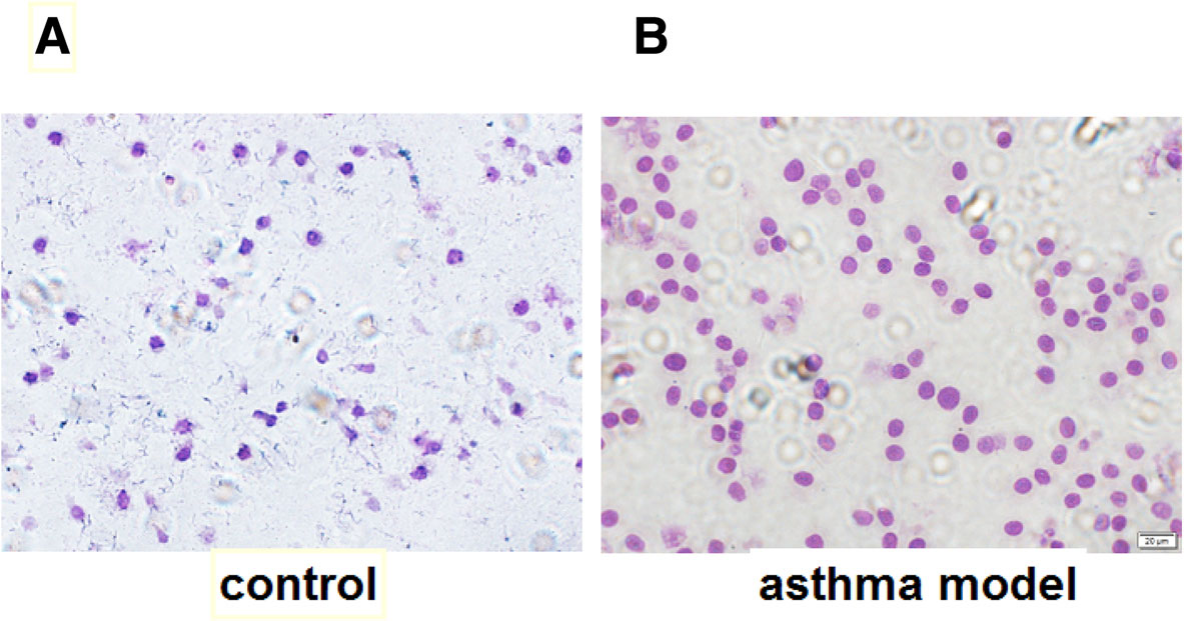
The comparison of alveolar lavage fluid flake. **a** The flake condition of the control group.**b** The flake condition of the asthma model group

The alveolar lavage fluid flake was shaken and stained, and the infiltration of eosinophils in the alveolar lavage fluid was observed. There was more infiltration of eosinophils in the asthma model group than the control so that the asthmatic rat model was successfully established. The asthmatic rats had behaved shortness of breath, irritability, piloerection and scratching their noses. The results are shown in Fig. 2a-b.

### Evaluation of notch signaling pathway inhibition

**Fig. 3:**
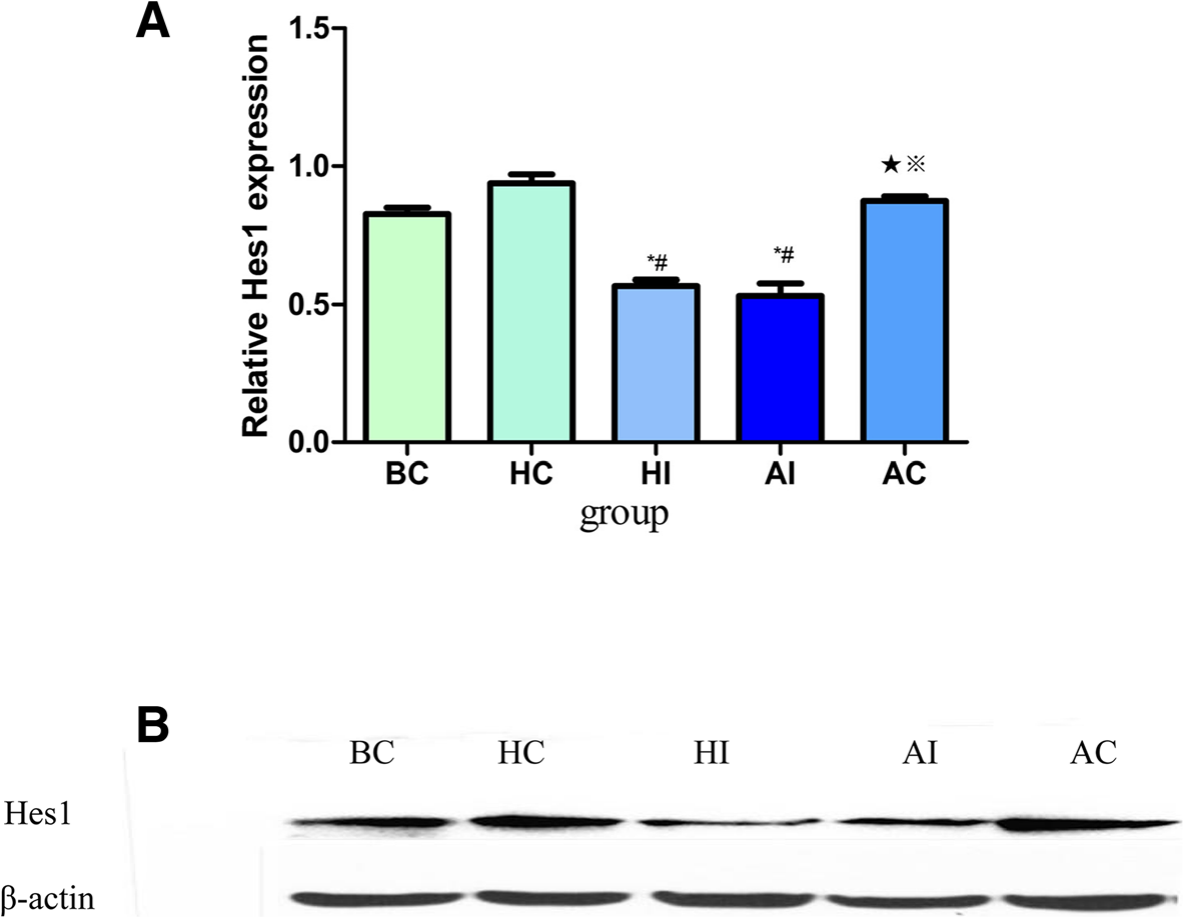
Western blot determination of proteins in the lung (*n* = 10 animals/each group). **a** The densitometric scans of Hes1/β-actin; **b** Western blot assay of Hes1 expression in the liver after DAPT treatment. The histogram represents the mean ± SD of the densitometric scans for protein bands from each group and normalized to β-actin. ^#^Statistically significant difference compared with HC (*p <* 0.05); ^★^Statistically significant difference compared with HI (*p <* 0.05); ^※^Statistically significant difference compared with AI(*p* < 0.05); ^*^ Statistically significant difference compared with BC (*p* < 0.05). Abbreviation: BC: blank control group; HC: healthy control group; HI: healthy pathway inhibition group; AC: asthma model control group; AI: asthma pathway inhibition group

To confirm that DAPT has inhibitory effects on Notch signaling pathway in rats, we performed western blots and found that the expression level of hes1 was significantly lower in the HI and AI groups than other three control groups (*p* < 0.05, Fig. 3a-b). Obviously, DAPT has already worked and the Notch signaling pathway has been successfully suppressed.

### Effect of PM_2.5_ on body weight in rats

In the first group of experiments, as shown in Table 1, there was no statistical difference in the body weight of rats after two acute PM_2.5_ exposures (*p* > 0.05), indicating that acute exposure was not effective in rats’ weight. In the second group of experiments, as shown in Table 2, after asthma model established, the weight of the BC group was significantly lower than other four groups (*p* < 0.05), indicating that asthma has a significant role in promoting weight gain in rats. After exposure to PM_2.5_, the body weights of the rats in the AL, AM and AH groups were significantly lower than the BC group (*p* < 0.05), indicating that exposure to PM_2.5_ could promote the weight loss in asthmatic rats. In the third group of experiments, at each stage of the experiment, there was no significant difference in body weight between the groups (*p* > 0.05), indicating that inhibition of Notch signaling pathway had no effect on body weight of rats, as shown in Table 3.

**Table 1:** Effects of exposure to PM_2.5_ on body weight in normal rats(g)

Group	Exposure time (day)		
	0	2	5
BC	216.57 ± 22.01	222.00 ± 26.20	223.50 ± 40.00
LD	218.14 ± 17.85	213.71 ± 21.88	201.71 ± 14.20
MD	227.57 ± 13.81	236.86 ± 16.70	216.71 ± 16.07
HD	213.14 ± 18.94	226.14 ± 19.40	198.60 ± 20.19

**Table 2:** Effects of exposure to PM_2.5_ on body weight after asthma model establishment in rats(g)

Group	Exposure time (week)		
	0	2	4
BC	216.57 ± 22.01	223.50 ± 40.00	283.00 ± 37.50
AC	221.00 ± 11.36	327.67 ± 47.78*	323.00 ± 48.17
AL	220.00 ± 18.54	282.89 ± 41.50*	272.33 ± 42.04*
AM	213.00 ± 15.80	276.00 ± 41.63*	245.67 ± 21.56*
AH	209.43 ± 10.05	282.00 ± 35.23*	264.83 ± 36.67*

*p < 0.05, compared with BC group

**Table 3:** Effects of exposure to PM_2.5_ on body weight after the Notch signaling pathway inhibition in rats(g)

Group	Exposure time (week)		
	0	2	4
BC	313.00 ± 55.71	362.80 ± 94.68	359.23 ± 100.84
HC	304.90 ± 56.90	361.20 ± 91.94	323.05 ± 79.41
HI	302.80 ± 47.61	378.60 ± 84.63	332.57 ± 75.98
AC	298.60 ± 35.60	349.10 ± 69.66	311.20 ± 56.69
AI	295.00 ± 36.70	359.00 ± 74.76	321.54 ± 71.11

Abbreviation: *BC* blank control group, *LD* low dose (PM_2.5_ 1.5 mg/kg) group, *MD* middle dose (PM_2.5_ 7.5 mg/kg) group, *HD* high dose (PM_2.5_ 37.5 mg/kg) group, *AC* asthma model control group, *AL* asthma model low dose (PM_2.5_ 1.5 mg/kg) group, *AM* asthma model middle dose(PM_2.5_ 7.5 mg/kg) group, *AH* asthma model high dose(PM_2.5_ 37.5 mg/kg) group, *HC* healthy control group, *HI* healthy pathway inhibition group, *AI* asthma pathway inhibition group

### Effect of PM_2.5_ on serum lipid levels in normal rats

**Fig. 4:**
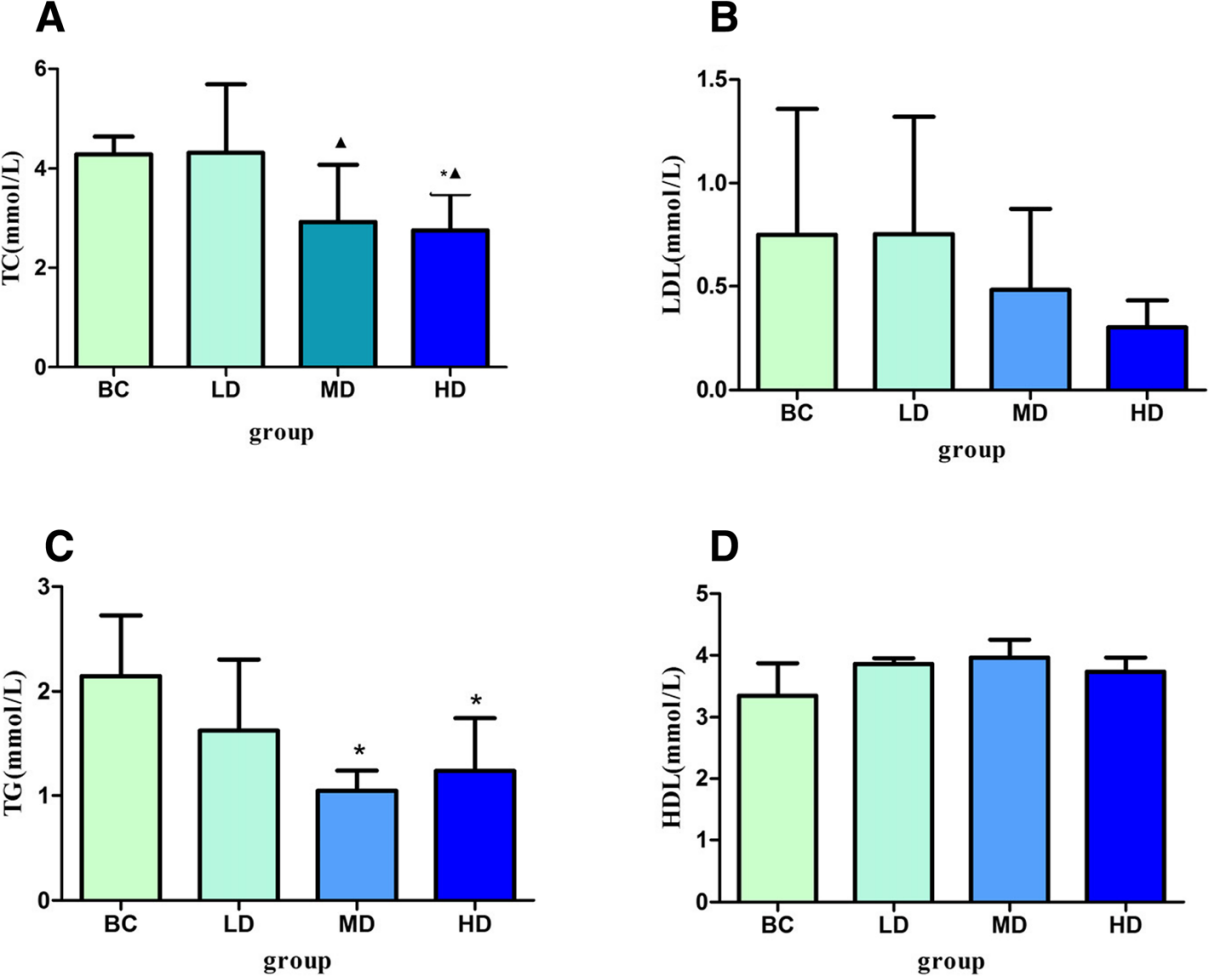
Effect of different doses of PM_2.5_ on serum lipid levels in normal rats (*n* = 6 animals/each group). **a** Total cholesterol (TC) level; **b** Low density lipoprotein cholesterol (LDL) level; **c** Triglyceride (TG) level; **d** High density lipoprotein cholesterol (HDL) level. ^▲^Statistically significant difference compared with LD (*p* < 0.05); ^*^ statistically significant difference compared with BC (*p* < 0.05). Abbreviation: BC: blank control group; LD: low dose (PM_2.5_ 1.5 mg/kg) group; MD: middle dose (PM_2.5_ 7.5 mg/kg) group; HD: high dose (PM_2.5_ 37.5 mg/kg) group

We directly administered different doses of PM_2.5_ to normal rats and tested lipid levels in rat serum. We found no statistical differences in serum LDL levels among different groups (*p* > 0.05, Fig. 4b). The serum TC levels in HD and MD groups were significantly lower than those in the LD group, while the TC levels in the HD group were significantly lower than those in BC group (*p* < 0.05, Fig. 4a). Compared with BC group, serum TG levels were significantly decreased in MD and HD groups (*p* < 0.05, Fig. 4c). In addition, serum HDL levels in the LD and MD groups were significantly higher than those in BC group (*p* < 0.05, Fig. 4d).

### Effect of PM_2.5_ on serum lipid levels in asthmatic rats

**Fig. 5:**
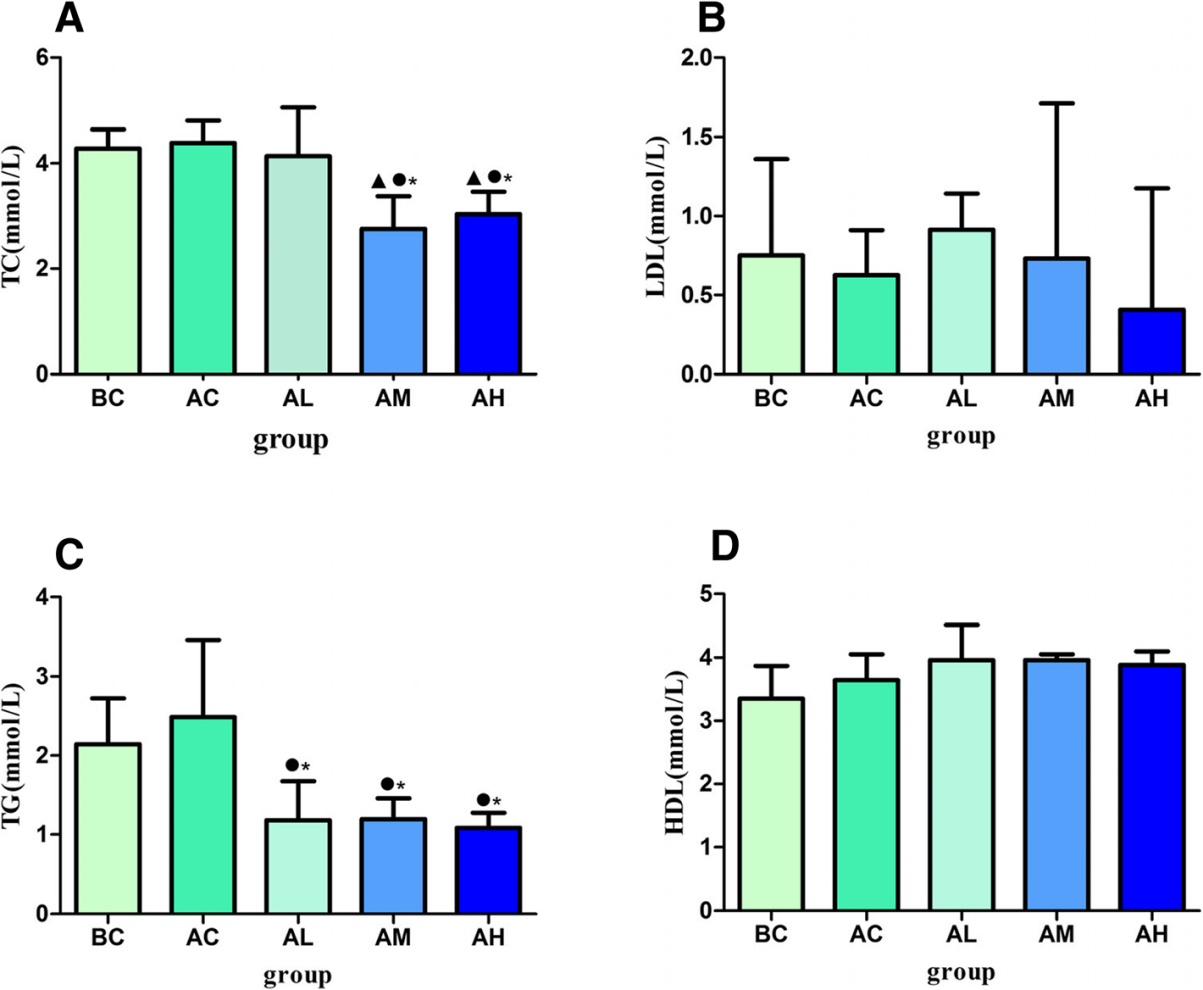
Effect of different doses of PM_2.5_ on serum lipid levels after asthma model establishment in rats (*n* = 8 animals/each group). **a** Total cholesterol (TC) level; **b** Low density lipoprotein cholesterol (LDL) level; **c** Triglyceride (TG) level; **d** High density lipoprotein cholesterol (HDL) level. ^●^Statistically significant difference compared with AC (*p* < 0.05); ^▲^Statistically significant difference compared with AL (*p* < 0.05); ^*^ statistically significant difference compared with BC (*p* < 0.05). Abbreviation: BC: blank control group; AC: asthma model control group; AL: asthma model low dose (PM_2.5_ 1.5 mg/kg) group; AM: asthma model middle dose (PM_2.5_ 7.5 mg/kg) group; AH: asthma model high dose (PM_2.5_ 37.5 mg/kg) group

After successful establishment of asthma model rats, different doses of PM_2.5_ were given to detect the lipid levels in rat serum. It was found that there was no significant difference in serum LDL levels among different groups (*p* > 0.05, Fig. 5b). Serum TC levels in AH and AM groups were significantly lower than those in AL, AC and BC groups (*p* < 0.05, Fig. 5a). Compared with BC and AC groups, serum TG levels in the AL, AM and AH groups were significantly decreased (*p* < 0.05, Fig. 5c). It was found that there was no significant difference in serum HDL levels among different groups (*p* > 0.05, Fig. 5d).

### Effect of PM_2.5_ on serum lipid levels in rats notch signaling pathway inhibited

**Fig. 6:**
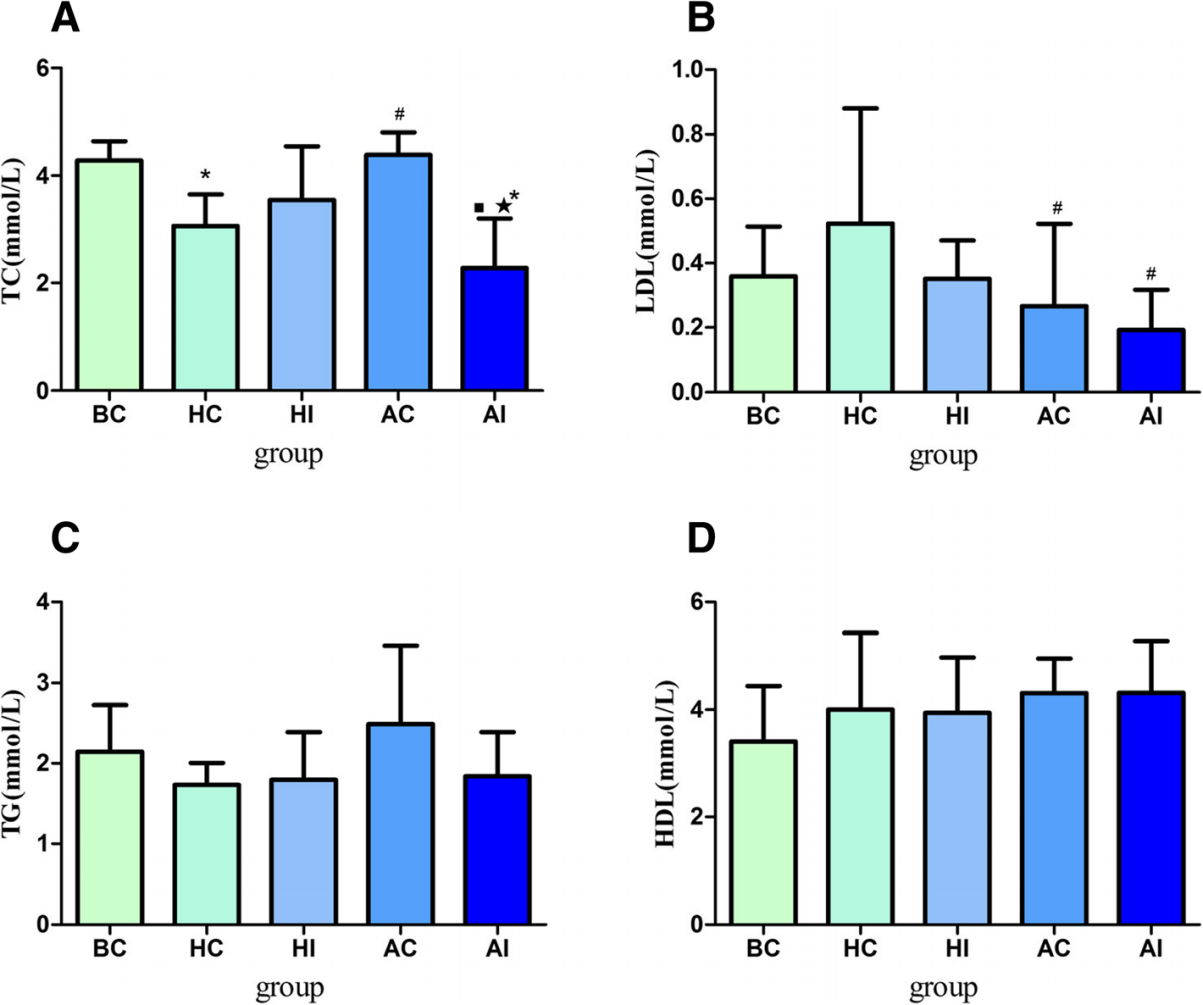
Effects of exposure to PM_2.5_ on serum lipid levels after the Notch signaling pathway inhibition in rats (*n* = 10 animals/each group). **a** Total cholesterol (TC) level; **b** Low density lipoprotein cholesterol (LDL) level; **c** Triglyceride (TG) level; **d** High density lipoprotein cholesterol (HDL) level. ^#^Statistically significant difference compared with HC (*p* < 0.05); ^★^Statistically significant difference compared with HI (*p* < 0.05); ^■^Statistically significant difference compared with AC (*p* < 0.05); ^*^ statistically significant difference compared with BC (*p* < 0.05). Abbreviation: BC: blank control group; HI: healthy pathway inhibition group; AC: asthma model control group; AI: asthma pathway inhibition group

We used DAPT to inhibit the Notch signaling pathway, administered 7.5 mg/kg of PM_2.5_ and detect the lipid levels in rat serum. We found no statistical difference in serum HDL levels b among different groups (*p* > 0.05, Fig. 6d). Compared with the AC, HI and BC groups, serum TC levels in the AI group were significantly decreased. The levels in the AC group were also significantly higher than the HC group (*p* < 0.05,Fig. 6a). Serum LDL levels were significantly lower in the AC and AI groups, compared with the healthy control group (*p* < 0.05,Fig. 6b). Meanwhile we found no statistical difference in serum TG levels among different groups (*p* > 0.05, Fig. 6c).

### Effect of asthma on serum lipid levels in rats exposed to PM_2.5_

**Fig. 7:**
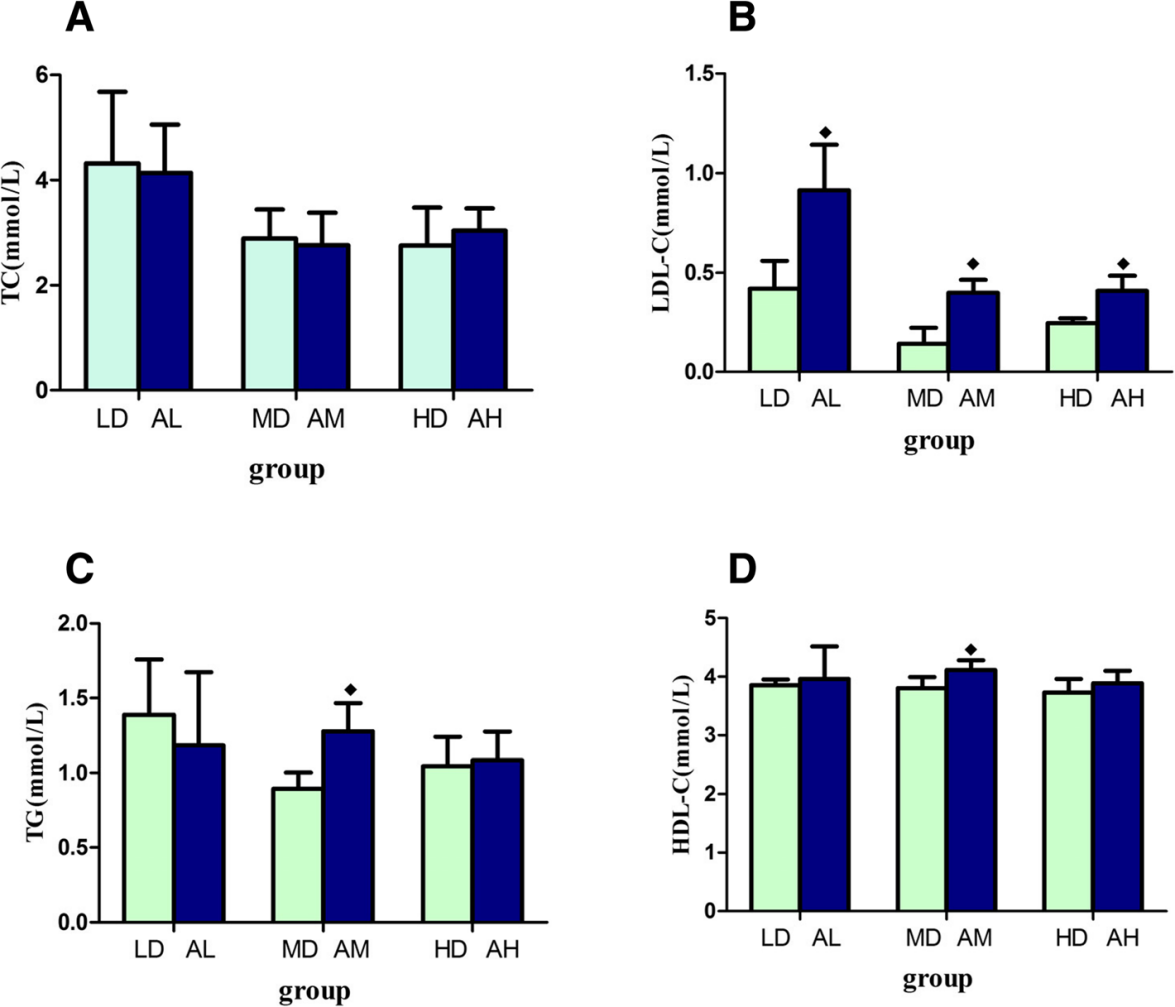
The effects of asthma on serum lipid levels in rats infected with PM_2.5_. **a** Total cholesterol (TC) level; **b** Low density lipoprotein cholesterol (LDL) level; **c** Triglyceride (TG) level; **d** High density lipoprotein cholesterol (HDL) level. ^◆^Statistically significant difference compared with direct exposure group (*p* < 0.05). Abbreviation: BC: blank control group; LD: low dose (PM_2.5_ 1.5 mg/kg) group; MD: middle dose (PM_2.5_ 7.5 mg/kg) group; HD: high dose (PM_2.5_ 37.5 mg/kg) group; AC: asthma model control group; AL: asthma model low dose (PM_2.5_ 1.5 mg/kg) group; AM: asthma model middle dose (PM_2.5_ 7.5 mg/kg) group; AH: asthma model high dose (PM_2.5_ 37.5 mg/kg) group

We chose the rats directly given PM_2.5_ as one group and the other group, which was given the same dose of PM_2.5_ for detection and comparison after asthma model establishment. We found that there was no statistical difference of serum TC levels between the two groups (*p* > 0.05, Fig. 7a). The serum levels of LDL in the asthma model group were significantly higher than that in the direct group, in the three dose groups (*p* < 0.05, Fig. 7b). At the same time, when the PM_2.5_ was 7.5 mg/kg, the serum levels of TG and HDL were higher in the model group than that in the direct group (*p* < 0.05, Fig. 7c and d).

## Discussion

Nowadays, using quartz fiber membrane is the most common method to collect atmospheric PM_2.5_. The ability of adsorption gas is better than other membrane filters, and its influence on the body is negligible after ultrasonic elution [25, 26]. In our vitro cell experiments, there was no statistically significant difference between the effect of filter control group and saline control group on the cells.

At present, we usually use the nasal instillation method for PM_2.5_ exposing. This method is required that the rats need be anesthetized first, and PM_2.5_ that is evenly distributed in physiological saline should be instilled into the rat respiratory tract by a micropipette. Due to the large size of rats, the tracheal opening is relatively difficult. After reading the literature [27] and a large number of pre-experiments, we chose four dose groups of 0, 1.5, 7.5, and 37.5 mg/kg, respectively.

Successful establishment of asthma model rats is the basis to this study. Therefore, we selected the most widely used chicken ovalbumin to induce the establishment of asthma model [28]. While, asthma is a chronic airway inflammatory disease characterized by eosinophils [29]. We observed that infiltration of eosinophils in the asthma model rats was increased by smearing of alveolar lavage fluid to determine the success of modeling.

It is well known that DAPT is a good Notch signaling pathway inhibitor [21], which can effectively knock out the notch signaling pathway. It is only a shallow study of the relationship between notch and lipid metabolism. To study the mechanism level further, we will consider combining Notch signaling pathway inhibitors and agonists to observe the effects of both on lipid metabolism in future studies. Masuko Katoh confirmed that Hes1 is a target gene of the Notch signaling pathway [30]. The expression of Hes1 is an important marker for the activation of Notch signaling pathway. We found that the expression level of hes1 was significantly lower in the healthy and asthma pathway inhibition groups than that in the other three groups. It suggested that the Notch signaling pathway was successfully suppressed.

By monitoring the weight of rats at different times, we found that acute PM_2.5_ exposing could not cause changes in body weight of rats and no organic changes had occurred in the organs of rats. However, after the establishment of asthma model, we found that the weight of rats in each dose group was higher than that of the control group, and it can be speculated that asthma could promote the occurrence of obesity [31]. After exposed to PM_2.5_, low, medium and high doses all aggravated symptoms such as wheezing, shortness of breath and irritability in the abdominal muscles of the asthmatic rats. The body weight of the three groups was significantly lower than the control group, suggested that PM_2.5_ could upregulate airway inflammation, damage the body and consume nutrients (such as fat, protein etc.) in asthmatic rats. At each stage of the third experiment, there was no significant difference in body weight among the rats in each group, indicating that inhibition of Notch signaling pathway had no effect on body weight of rats.

The principle of the dyslipidemia in cardiovascular disease has been extensively studied, but its role in lung-related diseases has been recently attracted attention. We can observe that rats exposed to PM_2.5_, the levels of TC and TG in the middle and high dose groups decreased significantly, while the levels of HDL in the low and middle dose groups increased significantly, indicating PM_2.5_ can promote the production of HDL, inhibit TC and TG. Therefore, we speculated that PM_2.5_ may enter the body from the bronchus and lungs, contact with and interact with lung epithelial cells and pulmonary macrophages to release a large number of cytokines and ROS [32], which could induce airway inflammation and oxidative damage. These ROS and the free radicals carried by the particles themselves enter the blood circulation through the blood-blood barrier, causing lipid peroxidation of the cells and then leading to dyslipidemia [33–35]. The mechanism of PM_2.5_ on blood lipid levels remains to be further studied.

A number of studies have shown that the increase in the concentration of PM_2.5_ promotes the prevalence of asthma and worsens the symptoms of asthmatic patients [36–38]. Therefore, we studied the blood lipid levels in asthma model rats infected PM_2.5_, and then tried to find a link between blood lipids and asthma. In the present study, it can be seen that the levels of TC and TG in the medium and high dose groups decreased significantly, while the HDL levels in the low and medium dose groups increased significantly. The imbalance of HDL in the body may be one of the causes of asthma [39]. At present, the research on the relationship between HDL and asthma is still in its infancy, and the research results are different. Recent studies found that LDL is associated with asthma and might be a potential risk factor for asthma [40]. Villeneuve PJ found LDL with pro-inflammatory effects could affect endothelial dysfunction, endothelial cell damage and promote the development of asthma [41]. Dyslipidemia can alter the innate and adaptive immune responses of the lungs and increase the lung’s susceptibility to pathogens [42]. Excessive cholesterol load can activate the innate immune system, a reaction that was only recently found in the lungs [43, 44]. Cholesterol can synthesize lipid rafts in the plasma membrane. Studies have found that cholesterol levels in lipid rafts are significantly lower and may play an important role in the pathogenesis of asthma [45]. Meanwhile, a large epidemiological survey showed that TC and TG levels were negatively correlated with the prevalence of asthma [41], which is consistent with our experimental results.

It has been experimentally confirmed that Notch signaling pathway plays a role in the regulation of asthma immunity and inflammation [45]. We used DAPT, a Notch signaling pathway inhibitor [46], to inhibit the release and release of Notch’s active structure, NICD, to observe its effect on serum lipid levels in asthmatic rats. The levels of serum TC and LDL in the asthma pathway inhibition group were significantly lower than those in the healthy group. However, the serum TC levels in the healthy pathway inhibition group were significantly higher than those in the blank control group. Therefore, we can conclude that inhibition of Notch signaling pathway can improve certain lipid levels in asthmatic rats. An emerging theme is the ability of Notch to respond to changes in the microenvironment, including glucose and lipid metabolites [45]. The Notch signaling pathway is complex and involves four Notch receptors and five ligands in the Jagged/Delta-like family. In fact, the C2 phospholipid recognition domain in the N-terminal region of Jag1 was recently revealed. This domain is also present in Dll1 and does not affect dimerization with the receptor, but it regulates the level of Notch activation [47]. C Bernsmeier found that Notch signaling plays a regulatory role in cholesterol and lipid biosynthesis, body weight and energy storage [48]. In addition, Onoyama found that the regulation of lipid metabolism has been attributed to F-box, which is a molecule that targets and degrades Notch [49].

## Conclusions

Exposure to PM_2.5_ can cause dyslipidemia in rats and aggravate the abnormal lipid levels in asthmatic rats. The Notch signaling pathway can regulate blood lipid levels in asthmatic rats.

## Data Availability

Datasets used in this study will be made available upon request.

## Abbreviations

AC: Asthma model control
AI: Asthma pathway inhibition
BC: Blank control
DMSO: Dimethyl sulfoxide
GSI: Gamma-secretase inhibitor
HC: Healthy control
HDL: High density lipoprotein
HI: Healthy pathway inhibition
LDL: Low density lipoprotein
NICD: Notch intracellular domain
PM_2.5_: Fine specific matter
SE: Standard error of mean
TC: Total cholesterol
TG: Triglycerides
